# A Single Institution Consensus on the Use of Sequential or Concurrent Hormonal Therapy for Breast Cancer Patients Receiving Radiation Therapy

**DOI:** 10.7759/cureus.555

**Published:** 2016-04-03

**Authors:** Matthew J Cecchini, Edward Yu, Brian P Yaremko, R Gabriel Boldt, Kylea Potvin, Tracy Sexton, David D'Souza, Muriel Brackstone, Michael Lock

**Affiliations:** 1 Department of Pathology, Schulich School of Medicine & Dentistry, Western University, London, Ontario, CA; 2 Department of Radiation Oncology, London Regional Cancer Program, London, Ontario, CA; Schulich School of Medicine & Dentistry, Western University, London, Ontario, CA.; 3 Department of Radiation Oncology, London Regional Cancer Program, London, Ontario, CA; 4 Schulich School of Medicine & Dentistry, Western University, London, Ontario, CA; 5 London Health Sciences Centre; 6 Department of Medical Oncology, London Regional Cancer Program, London, Ontario, CA; Schulich School of Medicine & Dentistry, Western University, London, Ontario, CA; 7 Department of Radiation Oncology, London Regional Cancer Program, London, Ontario, CA; Schulich School of Medicine & Dentistry, Western University, London, Ontario, CA; 8 Department of Radiation Oncology, London Regional Cancer Program, London, Ontario, CA; 9 Department of Surgical Oncology, London Regional Cancer Program, London, Ontario, CA; Schulich School of Medicine & Dentistry, Western University, London, Ontario, CA

**Keywords:** breast cancer, concurrent, sequential, hormone therapy, aromatase inhibitors, tamoxifen

## Abstract

Background and objectives: For hormone-sensitive breast cancers, treatment with breast-conserving surgery, tamoxifen, or aromatase inhibitors, along with adjuvant radiation, is the mainstay of therapy. The ideal timing of hormonal and radiation treatment is not well defined, and there is a significant degree of practice variability between concurrent and sequential treatment regimes. This variability can cause confusion amongst the clinical team resulting in contradictory recommendations, loss of patient trust, and the potential for missed initiation of hormonal therapy.

Methods: To address this question, a systematic review of the literature was conducted and presented to the breast cancer multidisciplinary team at the London Regional Cancer Center. A three-round modified Delphi method was used to obtain a consensus on a series of a priori determined statements.

Results: With the currently available evidence, the consensus was that hormonal therapy should be given sequentially after radiation. This will limit potential overlapping adverse effects between hormonal therapy and radiation that may decrease completion of treatment. The sequential approach has not been associated with any harm in clinical outcomes, and there is some suggestion of increased toxicity with concurrent use. However, in patients at high risk of distant recurrence, they felt it would be reasonable to consider concurrent treatment to avoid any delay in therapy.

Conclusion: The consensus of our institution to utilize a sequential approach will standardize the treatment decisions and reduce the risk of failing to initiate hormonal therapy. Despite the lack of level 1 evidence, the Delphi methodology did provide a high level of confidence for our group to choose the sequential approach. The consensus was developed after a review of the literature revealed that there was no clear superiority of one schedule over the other and evidence that concurrent treatment may increase adverse events.

## Introduction

Breast cancer is the leading cause of cancer in women in Canada with an estimated 24,000 new cases in 2014 [1]. Fortunately, there is a good prognosis for women with early stage disease with a survival of 95% at five years [2]. For women with cancers expressing either the estrogen receptor or the progesterone receptor, treatment with tamoxifen in premenopausal women or aromatase inhibitors in postmenopausal women is associated with an improvement in overall survival [3-4]. Breast-conserving surgery with lumpectomy and sentinel node biopsy is one of the mainstays of treatment. When breast-conserving surgery is combined with adjuvant radiation, similar rates of local recurrence and overall survival are observed as are seen with more radical surgery [5].

There is variation in the timing of radiation and hormonal therapy with a number of clinical studies aimed at addressing this question [6-12]. No difference in overall survival or local recurrence has been observed between the sequential vs. concurrent treatment regimes. However, combined hormonal therapy and radiation showed increased rates of breast and lung fibrosis. Therefore, with the current state, there remains some clinical uncertainty regarding the optimal timing of hormonal and radiation therapy.

## Materials and methods

The purpose of the consensus process was to develop recommendations that would be implemented successfully within a comprehensive cancer center by engaging all principle caregivers in breast cancer. A systematic review of the literature pertaining to the timing of hormone and radiation therapy was performed and presented in a separate article [13]. The working group established a search strategy and was conducted in consultation with a librarian trained in research methodology (GB). The results of this review were presented to the Breast Disease Site Team outlining the clinical evidence for sequential versus concurrent treatment approaches. Key questions and clinical outcomes, called consensus issues (CI), were identified a priori by a working group consisting of a medical oncologist (KP), resident (MC), and radiation oncologists (ML, EY). A modified Delphi protocol was used to obtain consensus regarding the optimal treatment regime.

### Consensus-based guideline development process

A modified Delphi protocol was used with a three-round Delphi approach [14]. This approach was chosen to formalize the process, obtain a conclusion from a paucity of literature, provide a forum for discussion regarding current and future practice, and provide the direct input of as many members in order to have the recommendations implemented uniformly. The consensus issues addressed the strength of evidence for and against sequential hormone and radiation use for 1) tamoxifen and aromatase inhibitors, 2) adverse effects, and 3) administrative outcomes. The Breast Cancer Disease Site Team members included nurses, social workers, geneticists, breast oncologists (surgeons, radiation, medical), radiologists, and pathologists (Table 1). The first round was a formal presentation of the data with an initial discussion of the evidence and practice. All rounds were not anonymous. The second round consisted of an email of the consensus issues with the presentation of the evidence for each CI. Voting occurred after these first two rounds. The final round consisted of a voting on each CI, followed by a presentation of the data once more, and a final vote with discussion. The CI were presented using a Likert scale with four levels, ranging from ‘strongly agree’ to ‘strongly disagree’. If greater than 65% of responses were ‘strongly agree’ or ‘agree’, then a consensus of ‘endorsed’ was considered obtained for that CI. The consensus of ‘not endorsed’ was considered achieved when greater than 65% of responses were ‘strongly disagree’ and ‘disagree’. All other statements were considered ‘equivocal’ and no endorsement could be made. The resulting recommendation was reviewed and approved by the multidisciplinary team.

**Table 1:** Breast Disease Site Membership by Medical Specialty

Medical Oncology	Radiation Oncology	Surgical Oncology	Pathology/Radiology/Genetics/Symptom Support Care	Research
Dr. Karin Hahn	Dr. David D’Souza	Dr. Muriel Brackstone	Dr. Alan Tuck	Dr. Jeff Chen
Dr. Diane Logan	Dr. Michael Lock	Dr. Steven Latosinsky	Dr. Anat Kornecki	Dr. Doug Hoover
Dr. Kylea Potvin	Dr. Francisco Perera	Dr. Ward Davies	Dr. Ben Nachum	Dr. Allison Allan
Dr. Ted Vandenberg	Dr. Nancy Read	Dr. Doug Quan	Dr. Giulio Muscedere	Dr. Ann Chambers
Dr. Jawaid Younus	Dr. Tracy Sexton	Dr. Chellappa Rajgopal	Dr. Peter Ainsworth	-
Dr. David Ballingall	Dr. Olga Vujovic	Dr. Elizabeth Saettler	Dr. Anita Singh	-
Dr. Catriona Andrews	Dr. Brian Yaremko	Dr. Leslie Scott	-	-
Dr. Mary Eisenhauer	Dr. Edward Yu	Dr. Kathy Pratt	-	-
Dr. Ken Yoshida	Dr. Jeff Cao	-	-	-
Dr. Brian Dingle	-	-	-	-

## Results

The search identified 39 studies relating either directly or indirectly on the issue of sequencing hormonal therapy and radiation in breast cancer patients. The MEDLINE search and results are described elsewhere [13], but included peer-reviewed literature from 1995 to 2015. Seven additional studies were found based on this initial search. No consensus statements, systematic reviews, or guidelines were found.

### Tamoxifen

No difference in survival or local recurrence has been identified for concurrent vs. sequential treatment with tamoxifen or aromatase inhibitors and adjuvant radiation [8-9, 11-12, 15]. The data for tamoxifen relies upon three retrospective studies with a combined 1,082 patients and follow-up between eight to 10 years for the individual studies [8-9, 15]. The length of follow-up was thought to be significant for oncologic outcomes, but there was some concern that, since some of the patients were treated over 30 years ago, the chemotherapy and radiation protocols the patients received were significantly different from modern treatment. Furthermore, the retrospective analysis introduced the possibility of bias to the study and, indeed, small differences were noted between the treatment groups in baseline characteristics. Despite the lack of strong data, the multidisciplinary team felt that there was sufficient evidence that the risk of delayed tamoxifen therapy for a sequential approach likely did not differ from the concurrent approach. Further, a randomized trial has been initiated to address the timing of tamoxifen with radiation [16]. The primary endpoint is the pulmonary fibrosis adverse event rate, but patients are being followed for other long-term oncologic outcomes and this study may provide further guidance when it is complete [16].

Summary: The group consensus was that the evidence was sufficient to conclude that sequential and concurrent schedules of tamoxifen treatment were equivalent in terms of survival and recurrence. 

### Aromatase inhibitors

In terms of aromatase inhibitors, there is less evidence in terms of outcome data for sequential versus concurrent treatment regimes [6, 10-12]. Four studies have addressed this question. The two studies that have addressed outcome were both retrospective in nature with limited follow-up between two and five years and only included 498 patients [11-12]. The patients were treated with more modern techniques in these studies. There was no difference noted in the outcome or local recurrence rates between the two groups in any of the four studies.

Summary: The consensus was that with short-term follow-up and despite some pre-clinical data, there is no evidence that the aromatase inhibitors sequence of treatment has an impact on survival or recurrence. 

### Adverse events

In terms of adverse events associated with the treatment regimes, no difference has been identified in studies directly comparing concurrent and sequential regimes. However, there is evidence that identifies increased rates of low-grade breast fibrosis [17-18] and lung fibrosis [19-21] in patients treated with combined radiation and tamoxifen compared to patients treated with radiation alone. The group acknowledged that the follow-up may be insufficient as trial follow-up ranged from about two to seven years. The findings in these studies are supported by animal models that have identified an increase in transforming growth factor beta (TGFb) associated with tamoxifen use, which increases the rates of fibrosis in irradiated tissues [22-23]. However, these effects were asymptomatic and detected only on serial imaging in the case of lung fibrosis and not associated with changes in cosmetic outcome in the breast.

Summary: The consensus was that there might be an increased risk of fibrosis with concurrent therapy. Further study is recommended, particularly with standardized cosmetic and cardiac outcome measures over long periods.

### Cardiac toxicity

Toxicity to the heart has been identified as an important adverse event of radiation to the chest [24]. While the majority of radiation for breast cancer is delivered as photon breast tangents with little posterior divergence, there can still be an appreciable dose that is delivered to the heart, especially in left-sided breast cancer. TGFb has been identified as an important mediator of fibrosis in the heart and the increased levels associated with tamoxifen use could increase the risk [25]. To date, this important question has not been addressed in clinical studies. However, given that younger premenopausal women receive tamoxifen, these women are likely to have significant life spans after treatment, in some cases, 30 years or more. Therefore, if there are increased rates of cardiac fibrosis, this may put them at higher risks of important late cardiac events and warrants further study. 

Summary: The consensus was that there may be an increased risk of fibrosis with concurrent therapy. Further study is recommended.

### Quality assurance issues

In the discussion, the Breast Multidisciplinary Team (MDT) members recognized practice variability, with some radiation oncologists using concurrent hormonal therapy and radiation and others utilizing a sequential approach (67%). The medical oncology team expressed some difficulty in discussing this issue with patients, given the variability in local radiation oncology practice. This was raised as a quality assurance issue as the variability in practice could increase the likelihood that initiation of hormonal therapy could be missed or delayed. Furthermore, patients may experience a perception that their care was not well coordinated or that they may not be receiving the best possible care. Compliance/adherence issues may have a greater impact on the outcome than the choice of the sequence. Non-adherence has been shown to increase death rates and utilization of health care resources [26-28]. In addition, the variation in adherence rates by specialty was raised with retrospective data indicating that non-adherence rates were 47% in radiation oncologists and 34% with medical oncologists (p < 0.001) [29].

Summary: The need for a single recommendation is necessary to provide consistent patient care and improve the use of medication with a survival advantage.

### Overlapping treatment complications and adherence to treatment

The group also discussed the anecdotal experience that combined hormonal therapy and radiation were often more symptomatically difficult for patients to tolerate. Often, patients receiving hormonal therapy with radiation would attribute symptoms, such as vasomotor symptoms, joint pain, or fatigue, to the radiation. This could often generate difficulties in completing radiation therapy, even though these symptoms are more likely to be attributed to the hormonal agents.

Summary: Sequential treatment is recommended to reduce the possibility that side effects of hormonal therapy would decrease the ability of patients to complete treatment.

### High-risk patients

The team brought up the subgroup of patients at high risk for recurrence and/or distant microscopic disease. In these patients, would starting hormonal therapy as soon as possible to avoid untreated out-of-field disease be better? The sequential approach in these patients may not be appropriate due to the delay of treatment for up to six weeks. Two counterarguments were raised based on the literature. First, there is a theoretical decrease in the efficacy of radiation in hormonally treated cells due to the antiproliferative effects that have been shown in pre-clinical studies when concurrent treatment is given [30-32]. The group recognized that these studies were variable and contradictory. Indeed, the decrease in efficacy has not been observed clinically, and some studies have identified a synergistic increase in apoptosis associated with combined radiation and hormonal therapy [33-34]. Second, studies comparing sequencing regimens often included high-risk patients, especially node-positive disease cases. Also, patients with low-risk disease may be most likely to benefit from hormonal therapy as no other systemic treatment is given. The group stressed the importance of documentation and communication amongst health care providers and with the patient if there was to be a deviation of practice. 

Summary: In certain patients with a high risk of distant disease, it is not unreasonable to treat with concurrent hormonal agents and radiation.

## Discussion

Limitations of this study include the fact that many of the statements are not based on high-level evidence with large gaps in knowledge. However, the purpose of this paper was to obtain a consensus when there is insufficient evidence using a modified Delphi technique. This technique allows one to obtain a consensus in an area that lacks clear-cut evidence by synthesizing the knowledge and experience of experts. A further limitation is that this is a single center consensus and may be biased to local institutional practices. However, this, to our knowledge, represents the first Canadian Cancer Center to develop a uniform evidence-based approach to the sequence of hormonal and radiation therapy. The majority of the respondents were medical or radiation oncologists, and the conclusions may not reflect other experts in the field, such as translational scientists and radiobiologists, who were not included in the survey. This work represents a real-world scenario where the principle prescribers are medical or radiation oncologists who must make decisions routinely on this subject based on insufficient evidence.

## Conclusions

In consideration of the current state of evidence, the Breast MDT at the London Regional Cancer Program has developed a consensus agreement for the timing of hormonal therapy and radiation. Given that no harm was associated with delaying hormonal therapy during the course of radiation, the group will support the sequential model of hormonal therapy and radiation. This provides a consistency in treatment recommendations that will limit the chance that patients will miss the initiation of hormonal therapy and provide a consistent message to the patient from their various caregivers. Further, this schedule will limit the potential increase in fibrosis that may be associated with concurrent treatment regimes. In addition, patients may tolerate the sequential approach better as they would only have side effects associated with one treatment modality at a time. However, in patients with high clinical suspicion for distant or high-risk disease, it would also be reasonable to use hormonal agents in a concurrent fashion due to the quality of available data. Lastly, a consistent practice would provide a means to assess patients for future study.
